# Contrasting Molecular Imaging Findings in Hepatocellular Carcinoma Using Dual Positron Emission Tomography Modalities

**DOI:** 10.7759/cureus.75097

**Published:** 2024-12-04

**Authors:** Ahmed Abdlkadir, Obayda Rabei, Ula Al-Rasheed, Akram Al-Ibraheem

**Affiliations:** 1 Nuclear Medicine and PET/CT, King Hussein Cancer Center (KHCC), Amman, JOR

**Keywords:** 18f-fdg, 68ga-fapi, hcc, isometabolism, pet/ct, photopenia

## Abstract

Fibroblast activation protein (FAPI) has been recently incorporated as a molecular imaging radiotracer for the evaluation of epithelial neoplasms that support or complement the role of [^18^F]Fluorodeoxyglucose ([^18^F]FDG) in many cancer subtypes since its development. Both radiotracers have been shown to have diagnostic, prognostic, and predictive value for several neoplasms. Herein, we present a 73-year-old male patient with a complex medical and oncological history who was recently diagnosed with hepatocellular carcinoma (HCC). Both [^18^F]FDG and [^68^Ga]Ga-FAPI-04 revealed distinct molecular imaging characteristics. A large segment 8 hepatic mass was observed via [^18^F]FDG positron emission computed tomography (PET/CT), whereas [^68^Ga]Ga-FAPI PET/CT revealed a large hepatic mass confined to segment 8, exhibiting complete photopenia. Overall, T2N0M0 stage II HCC disease was affirmed. The patient underwent two rounds of transarterial chemoembolization, which resulted in partial disease control and symptomatic control. To our knowledge, this case represents the first imaging instance to capture such unique molecular characteristics of HCC via a dual PET/CT approach composed of [^18^F]FDG and [^68^Ga]Ga-FAPI radiotracers.

## Introduction

Hepatocellular carcinoma (HCC) is the most prevalent primary liver malignancy, accounting for approximately 85% of cases [1]. According to the Global Cancer Observatory (GLOBOCAN) 2022 estimates, HCC ranks as the sixth most frequently diagnosed cancer and the third leading cause of cancer-related mortality worldwide [1]. In 2022, the global incidence was estimated at 905,700 newly diagnosed cases, with a mortality rate of 830,200 [2]. Cirrhosis remains the primary risk factor for HCC, with advanced age, male gender, and viral hepatitis B and C infections also contributing significantly to its development [1]. Unlike other solid tumors, HCC diagnosis in cirrhotic patients is often non-invasive, relying primarily on diagnostic imaging [3]. Histological confirmation is only necessary in cases of atypical radiological presentation and in non-cirrhotic patients [3].

Recent advancements in molecular imaging techniques have led to increased investment in and utilization of [^68^Ga]Ga-Fibroblast activation protein inhibitor ([^68^Ga]Ga-FAPI) positron emission tomography/computed tomography (PET/CT) as a novel diagnostic agent for HCC [4]. Recent studies have demonstrated the superior diagnostic reliability of this innovative agent compared to the traditional [^18^F]Fluorodeoxyglucose ([^18^F]FDG) radiotracer, primarily due to its higher tumor-to-background ratio [5,6]. This enhanced performance is attributed to the abundant expression of FAPI derived from cancer-associated fibroblasts in the tumor microenvironment [4]. Despite its specific and unique uptake mechanism, this radiotracer is not without limitations in cancer imaging, as instances of false-positive and false-negative findings have emerged in recent years with increased clinical application [7].

This case report presents a unique instance wherein a dual PET/CT approach was employed for an HCC patient, revealing a FAPI-photopenic, FDG-isometabolic HCC mass. Through this case, we aim to discuss potential etiologic factors underlying this distinctive uptake pattern, drawing upon previous studies in molecular imaging and immunohistochemistry.

## Case presentation

We present the case of a 73-year-old male with a complex past history. His oncological history began 37 years ago with an incidental diagnosis of stage II pancreatic ductal adenocarcinoma treated with 5-fluorouracil, doxorubicin, and mitomycin (FAM) chemotherapy regimen. Fourteen years ago, he was diagnosed with stage III diffuse large B-cell lymphoma (DLBCL), which was successfully treated with six cycles of rituximab, cyclophosphamide, doxorubicin, vincristine, and prednisone (R-CHOP) protocol. The patient's history also includes ventricular arrhythmia managed by a cardiac defibrillator and successfully treated hepatitis C. After a decade of post-hepatitis C follow-ups, he discontinued visits due to consistently unremarkable results.

Recently, he presented with right upper quadrant abdominal pain, prompting a comprehensive evaluation. Laboratory tests revealed mild thrombocytopenia, elevated direct bilirubin, and elevated hepatic transaminases (Table 1).

**Table 1:** Baseline biochemical profile

Lab Test	Result	Normal Reference	Units
Hemoglobin	13.1	13-18	g/dL
Leukocytes	5200	4000-11000	/µL
Platelets	111	150-400	/µL
Urea	36.5	17-45	mg/dL
Creatinine	0.8	0.5-1.1	mg/dL
Total Bilirubin	0.5	0.3-1	mg/dL
Direct Bilirubin	0.37	0.1-0.3	mg/dL
Alanine Aminotransferase	147	7-47	U/L
Aspartate Aminotransferase	77.1	8-48	U/L
Alkaline Phosphatase	128	37-130	U/L
Albumin	4.3	4-5.1	g/dL
Fasting Blood Sugar	91.7	< 110	mg/dL
Uric Acid	5.1	2.6-8	mg/dL
Sodium	139.5	135-145	mmol/L
Potassium	4.6	3.6-5.2	mmol/L
Chloride	104.1	97-110	mmol/L
Calcium	9.4	8.8-10.2	mg/dL
Magnesium	2.03	1.7-2.4	mg/dL
Phosphorus	3.03	2.5-4.5	mg/dL
Alpha-Fetoprotein	1.9	< 7	ng/mL

**Figure 1:**
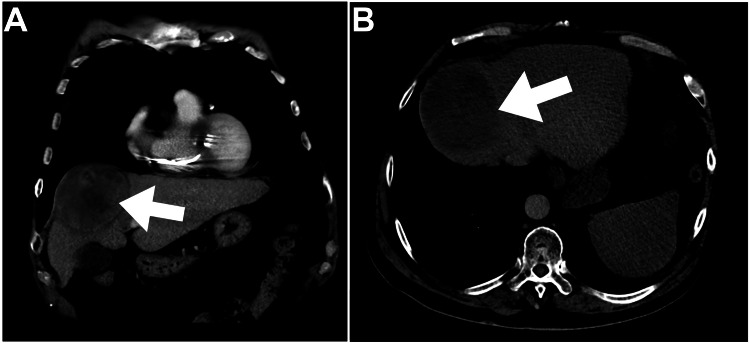
Baseline abdominal CT Coronal, and axial hepatic views of abdominal CT depicted a large 7.5 cm segment VIII hepatic mass (arrows).

An abdominal CT scan identified a large, well-defined subcapsular segment VIII liver lesion (Figure 1, arrows), classified as Liver Imaging Reporting and Data System (LI-RADS) 5.

**Figure 2:**
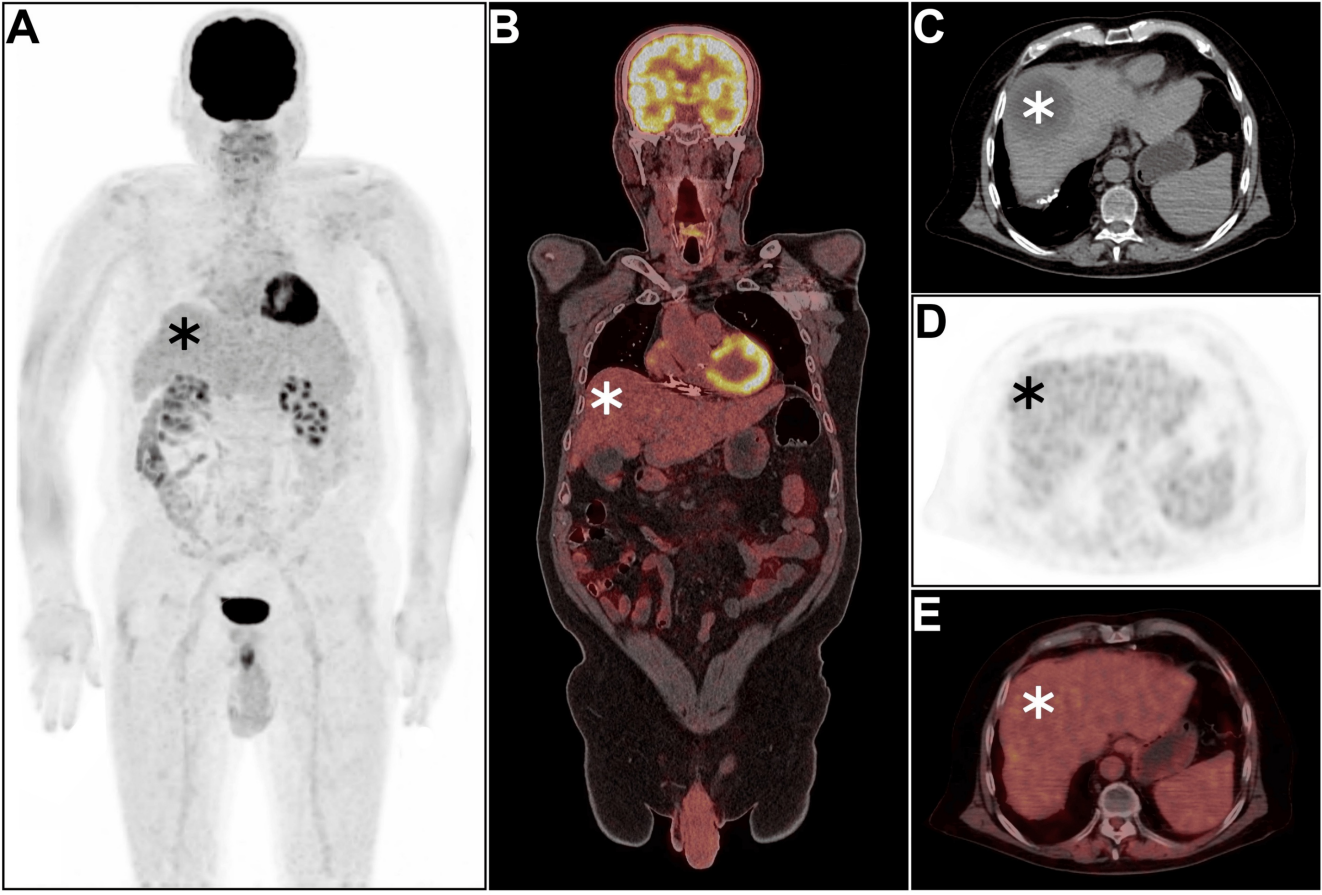
Baseline [18F]FDG PET/CT (A-E) Maximum intensity projection [^18^F]FDG image, fused coronal, and axial PET/CT views depicted the hepatocellular carcinoma lesion as an isometabolic segment VII hypodensity with otherwise unmarkable scan elsewhere (asterisks). FDG: Fluorodeoxyglucose

In addition, [^18^F]FDG PET/CT was offered for comprehensive oncologic evaluation (Figure 2). The scan confirmed the segment VIII hepatic lesion which was isometabolic and was unremarkable for any significant hypermetabolic lesions (Figure 2, asterisks).

**Figure 3:**
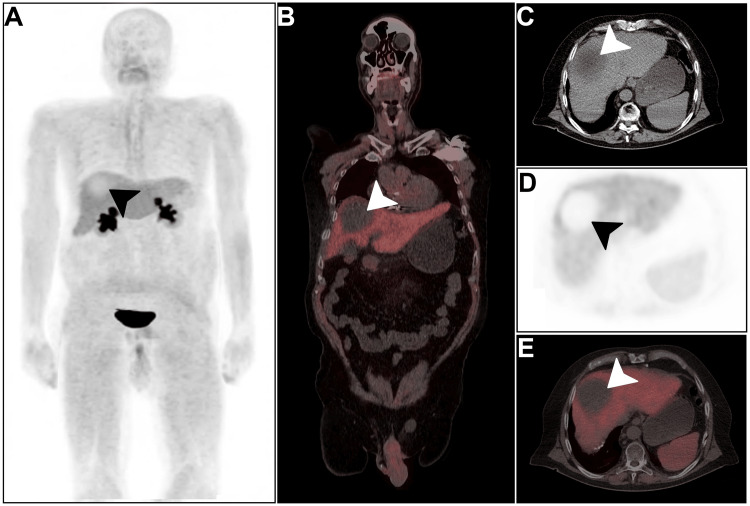
Baseline [68Ga]Ga-FAPI PET/CT (A-E) Maximum intensity projection [^68^Ga]Ga-FAPI, fused coronal, and axial PET/CT views depicted the hepatocellular carcinoma lesion as photopenic segment VII hypodensity with otherwise unmarkable scan elsewhere (arrowheads). FAPI: Fibroblast activation protein inhibitor

A multidisciplinary tumor board discussion recommended [^68^Ga]Ga-FAPI PET/CT, which revealed photopenic segment VIII liver lesion (Figure 3, arrowheads). Interestingly, no [^68^Ga]Ga-FAPI-avid lesion was depicted locally or elsewhere. A definitive diagnosis of HCC was established based on radiographic findings and clinical presentation.

**Figure 4:**
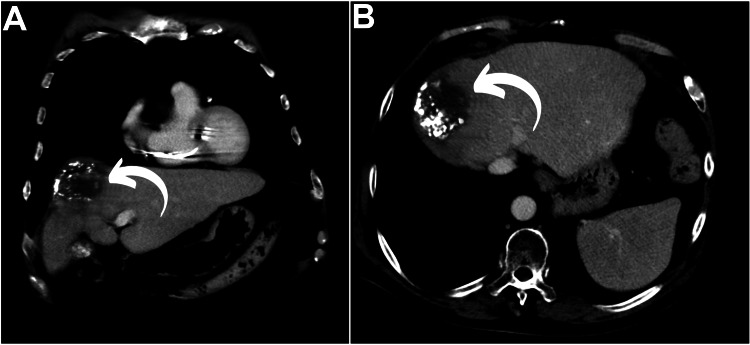
Follow-up abdominal CT (A, B) Partial tumor shrinkage and interval coalification were observed following transretinal chemoembolization (curved arrows).

Overall, T2N0M0 third primary neoplasm was affirmed and staged as stage II HCC. The patient underwent two sessions of trans-arterial chemoembolization (TACE) with 90 mg of doxorubicin and 2-3 ml of lipiodol, six months apart. Post-therapeutic evaluation with abdominal contrast-enhanced CT showed significant shrinkage of the liver mass (Figure 4, curved arrows), indicating a partial response, along with resolved abdominal pain and normalization of hepatic transaminases and direct bilirubin.

The patient will continue to receive regular oncologic care at our center, with further management decisions to be made in subsequent multidisciplinary discussions.

## Discussion

This case presents the complex medical and oncological journey of a patient recently diagnosed with a third primary neoplasm. A recent comparative study highlighted the poor survival metrics associated with multiple primary malignancies [8]. This prognosis is further compounded by the financial and psychological burden of disease discovery, prolonged follow-up, and multifaceted testing [8]. Recently, the adoption of dual PET/CT modalities has demonstrated utility in various clinical and oncological contexts [9].

The primary HCC was visualized by both modalities with contrasting molecular imaging patterns. On [^18^F]FDG PET/CT, the mass appeared to be isometabolic and indistinguishable from the surrounding liver parenchyma, except when it was reviewed on plain CT images. This is the metabolic pattern that typically occurs in well-differentiated HCC [10]. While this may be considered a limitation of [^18^F]FDG due to potential false negatives, it still provides vital predictive insights into tumor properties, especially in HCC, where radiologic diagnosis in cirrhotic patients is the reference standard. This is mainly because [^18^F]FDG PET/CT metabolic uptake has shown a substantial correlation with tumor aggressiveness and grade [11].

Examination of the [^68^Ga]Ga-FAPI PET/CT images revealed a photopenic pattern at the site of the primary HCC. Previous reports of [^68^Ga]Ga-FAPI photopenia have focused on the context of tumor necrosis [12]. However, tumor necrosis is unlikely to be the etiologic factor in this case, as it would result in matching photopenia in both [^18^F]FDG and [^68^Ga]Ga-FAPI radiotracers [13]. The photopenic [^68^Ga]Ga-FAPI HCC pattern may be attributed to inactive fibroblasts in early cancer stages [14]. Early stages of HCC involve a latency period during which fibroblasts experience a reversible shift between quiescent and normally activated states in an effort to evade immune system surveillance and damage [14]. While this phenomenon has not been extensively reported in [^68^Ga]Ga-FAPI PET/CT within the molecular imaging domain, previous immunohistochemistry research has revealed that HCC can exhibit FAP-immunostain negativity in 20% of cases [15]. A recent meta-analysis examining the diagnostic performance of FAPI PET/CT in primary hepatic neoplasms reported a pooled sensitivity of 94.3% and a pooled specificity of 89.3%. Consequently, there is a potential for false-negative results to arise in the assessment of HCC [16].

## Conclusions

In our case, both [^18^F]FDG and [^68^Ga]Ga-FAPI PET/CT were employed, revealing distinct molecular imaging characteristics. Although both modalities yielded false-negative results, the [^18^F]FDG isometabolic pattern suggested a low-grade HCC. Conversely, the photopenic HCC pattern observed on [^68^Ga]Ga-FAPI PET/CT was indicative of early-stage disease. Both scans provided valuable morphologic characterization of the HCC lesion, which is essential for definitive diagnosis. To our knowledge, this case represents the first documented instance of a photopenic HCC lesion captured using [^68^Ga]Ga-FAPI PET/CT. Consequently, nuclear medicine physicians should be aware of this potential limitation when evaluating HCC patients with [^68^Ga]Ga-FAPI PET/CT.
